# Analysis of the bacterial and fungal populations in South African sorghum beer (*umqombothi*) using full-length 16S rRNA amplicon sequencing

**DOI:** 10.1007/s11274-023-03764-4

**Published:** 2023-10-21

**Authors:** Edwin Hlangwani, Adrian Abrahams, Kedibone Masenya, Oluwafemi Ayodeji Adebo

**Affiliations:** 1 Food Innovation Research Group, Department of Biotechnology and Food Technology, Faculty of Science, University of Johannesburg, P.O. Box 17011, Doornfontein Campus, Johannesburg, South Africa; 2 Department of Biotechnology and Food Technology, Faculty of Science, University of Johannesburg, P.O. Box 17011, Doornfontein Campus, Johannesburg, South Africa; 3 Neuroscience Institute, University of Cape Town, Private Bag X3, Rondebosch, Cape Town, 7701 South Africa

**Keywords:** Opaque beer, Lactic acid bacteria, Microbial diversity, Next-generation sequencing

## Abstract

There is a need to profile microorganisms which exist pre-and-post-production of *umqombothi*, to understand its microbial diversity and the interactions which subsequently influence the final product. Thus, this study sought to determine the relative microbial abundance in *umqombothi* and predict the functional pathways of bacterial and fungal microbiota present. Full-length bacterial 16S rRNA and internal transcribed spacer (ITS) gene sequencing using PacBio single-molecule, real-time (SMRT) technology was used to assess the microbial compositions. PICRUSt2 was adopted to infer microbial functional differences. A mixture of harmful and beneficial microorganisms was observed in all samples. The microbial diversity differed significantly between the mixed raw ingredients (MRI), customary beer brew (CB), and optimised beer brew (OPB). The highest bacterial species diversity was observed in the MRI, while the highest fungal species diversity was observed in the OPB. The dominant bacterial species in the MRI, CB, and OPB were *Kosakonia cowanii*, *Apilactobacillus pseudoficulneus*, and *Vibrio alginolyticus*, respectively, while the dominant fungal species was *Apiotrichum laibachii.* The predicted functional annotations revealed significant (*p* < 0.05) differences in the microbial pathways of the fermented and unfermented samples. The most abundant pathways in the MRI were the branched-chain amino acid biosynthesis super pathway and the pentose phosphate pathway. The CB sample was characterised by folate (vitamin B_9_) transformations III, and mixed acid fermentation. Biotin (vitamin B_7_) biosynthesis I and l-valine biosynthesis characterised the OPB sample. These findings can assist in identifying potential starter cultures for the commercial production of *umqombothi*. Specifically, *A. pseudoficulneus* can be used for controlled fermentation during the production of *umqombothi*. Likewise, the use of *A. laibachii* can allow for better control over the fermentation kinetics such as carbohydrate conversion and end-product characteristics, especially esters and aroma compounds.

**Supplementary Information:**

The online version contains supplementary material available at 10.1007/s11274-023-03764-4.

## Introduction

*Umqombothi* is a sorghum-based beer with a creamy constituency, an opaque pinkish colour, and a sour taste (Adekoya et al. 2018a). Due to its availability, affordability, and cultural importance, *umqombothi* is enjoyed by low-income populations in the rural and semi-urban areas of South Africa (Hlangwani et al. 2020) and is of significant economic importance. The beer is generally considered safe for human consumption when prepared under standard fermentation conditions, although microbial contamination could pose potential safety challenges and poor-quality characteristics (Ikalafeng 2008). The opaque beer is often prepared in-home or market settings, where poor handling conditions can lead to contamination. The source of contamination can be the raw materials or utensils used in the brewing process (Lues et al. 2009). Pathogenic and spoilage microbes belonging to the genera *Bacillus, Escherichia, Proteus, Staphylococcus, Streptococcus, Aspergillus, Fusarium, Penicillium, Rhizopus* have been reported in *umqombothi* (Katongole 2008). These microbial contaminants in traditional African beverages often lead to turbidity problems, flavour changes, reduced fermentation and yeast performance, aroma defects, and batch contamination, subsequently decreasing consumer confidence and leading to potential financial losses (Obi 2017).

Thus, the determination of the microbial profile of a product such as *umqombothi* can be an important step in ensuring the product’s quality and safety meet the minimum regulatory requirements for consumer products. Traditionally, the profiling of microbial communities has been based on culture-dependent methods, although these have drawbacks, as not all microorganisms are culturable on standard laboratory growth media. This inefficacy of culture-dependent techniques has led to the use of next-generation sequencing (NGS), a technique that provides the possibility of determining mixed microbial communities in a sample (Yap et al. 2022). To date, several NGS platforms have been developed, and are used depending on the application. Commonly used NGS platforms include Oxford Nanopore, Illumina, 454, PacBio, and Ion Torrent (Meera Krishna et al. 2019). Illumina is currently the most widely used NGS platform (Hodzic et al. 2017). However, the platform possesses limitations such as the potential for base-composition bias, loss of some information from original full-length transcripts, a short read length (25 to 300 bases), and the requirement for polymerase chain reaction (PCR) amplification of multiple DNA templates before sequencing (Cui et al. 2020).

Due to technical constraints, researchers must choose the most effective target regions to identify taxa from full-length 16S rRNA gene sequences containing nine hypervariable regions (V1–V9) as phylogenetically informative markers (Kim et al. 2011; Yang et al. 2016). Therefore, long-read sequencing of the 16S rRNA gene is a promising approach for providing species-level analysis of bacterial communities (Johnson et al. 2019). A similar approach is applicable in targeting the internal transcribed spacer (ITS) region which has the highest probability of successful identification for the broadest range of fungi (Bradshaw et al. 2023). This region has the most clearly defined barcode gap between inter-and-intraspecific variation (Yang et al. 2016). PacBio can generate reads with a median length of 8–10 kb, up to as long as 100 kb, to circumvent these limitations (Loman et al. 2015). For this study, PacBio SMRT (Single Molecule, Real-Time) technology was used to characterise both bacterial and fungal compositions in *umqombothi.* In addition, functional annotations of the opaque beer samples were assessed using PICRUSt2.

## Methodology

### Traditional beer (*umqombothi*) brewing process

The brewing process followed a method described by Hlangwani et al. (2021a), whereby 500 g of King Korn malted sorghum (Tiger Brands, Johannesburg, South Africa) was mixed with 1000 g of White Star maize meal (Pioneer Foods, Paarl, South Africa) in a sterile 10 L bucket filled with 7 L sterile tap water. The mixture was gently stirred, covered, and incubated (Labcon, Chamdor, South Africa) at 25 °C for 24 h to sour. The optimised beer brew (OPB) was prepared following the optimal production parameters generated by the optimisation model in a preceding study (Hlangwani et al. (2021a). The mixed ingredients were then cooked for 1.1 h at 95 °C, and the mixture was allowed to cool to 25 °C. To the cooled porridge, 500 g of King Korn malted sorghum (Tiger Brands, Johannesburg, South Africa) was added and homogenised with gentle stirring. The mixture was then fermented at 29.3 °C for 25.9 h to obtain the OPB. In contrast, the customary beer brew (CB) was prepared following the traditional production method highlighted in Hlangwani et al. (2021b). The mixed ingredients were cooked for 30 min at 95 °C and the mixture was allowed to cool to 25 °C. To the cooled porridge, 500 g of King Korn malted sorghum (Tiger Brands, Johannesburg, South Africa) was added and homogenised with gentle stirring and the subsequent mixture was then fermented at 25 °C for 24 h. The mixed raw ingredients (MRI) were prepared by mixing 500 g of King Korn malted sorghum (Tiger Brands, Johannesburg, South Africa), 1000 g of White Star maize meal (Pioneer Foods, Paarl, South Africa), and 7 L of sterile tap water. The physicochemical properties of the respective samples were previously investigated (Hlangwani et al. 2021b) and are presented in Table 1.

**Table 1:** Physicochemical properties of mixed raw ingredients and *umqombothi* types (Hlangwani et al. 2021b)

Sample	Alcohol (°P)	TSS (g/100 g)	TTA (% lactic acid)	pH	Viscosity (cm/min)
MRI	10.47 ± 0.21^a^	10.30 ± 0.30^a^	0.50 ± 0.03^a^	4.60 ± 0.20^c^	12.83 ± 0.29^c^
CB	11.33 ± 0.21^b^	10.90 ± 0.10^b^	0.57 ± 0.02^b^	4.23 ± 0.02^b^	16.83 ± 0.76^b^
OPB	13.63 ± 0.12^c^	13.33 ± 0.21^c^	0.68 ± 0.02^c^	3.27 ± 0.03^a^	11.50 ± 0.87^a^

*CB* customary brew, *MRI* mixed raw ingredients, *OPB* optimized brew, *TSS* total soluble solids, *TTA* total titratable acidity. Each value is a mean ± standard deviation of triplicates

*Each value is a mean of triplicates ± SD of triplicates. Means with no common letters within a row significantly differ (*p* < 0.05)

### Microbial community profiling of *umqombothi*

#### DNA isolation

A modified cetyltrimethylammonium bromide (CTAB) extraction method was used to isolate the DNA (Tamari et al. 2013). Three sterile conical tubes were each used to suspend 1 g of freshly prepared optimised beer brew (OPB), customary beer brew (CB), and mixed raw ingredients (MRI) in 800 µL prewarmed (60 °C) CTAB extraction buffer and then incubated for 1 h. Thereafter, 800 µL chloroform:isoamyl alcohol (24:1) was added and the tubes were gently inverted 4 times to mix. The samples were centrifuged (Eppendorf, Hamburg, Germany) at 3 000 × *g* for 10 min. The aqueous phase was transferred to sterile microtubes, and 1 µL RNase (Sigma-Aldrich, Missouri, USA) was added to each microtube and incubated for 30 min at 37 °C. Subsequently, 600 µL of isopropanol was added to each microtube and the resulting mixture was gently mixed. The samples were left at room temperature for 4 h to precipitate. The samples were then centrifuged (Eppendorf, Hamburg, Germany) at 3000 × *g* for 20 min to form a pellet. Thereafter, the supernatant was removed, and the pellet was washed three times with cold (–4 °C) 70% ethanol and then centrifuged (Eppendorf, Hamburg, Germany) three times at 3000 × *g* for 10 min to remove excess ethanol. The microtubes were left open and allowed to air-dry under a laminar flow (Esco Micro Pte. Ltd., Changi, Singapore) at room temperature for 30 min to dry the pellets. Lastly, the pellets were resuspended in sterile distilled water and stored at –20 °C for subsequent analysis.

#### Deoxyribonucleic acid (DNA) quantification

The quality and quantity of the DNA were determined using a spectrophotometer at a wavelength of 200–400 nm (Implen GmbH, Munich, Germany). The instrument was calibrated by placing a drop of sterile distilled water on the instrument well before use.

#### Data sequencing and analysis

##### PCR amplification of the 16S rRNA gene

The polymerase chain reaction (PCR) was performed using a procedure by Klindworth et al. (2013). Primer pairs used were: 5${\prime}$-CCTACGGGNGGCWGCAG-3′ and 5′-GACTACHVGGGTATCTAATCC-3′ (Schloss et al. 2016). The primers targeted V1–V9 hypervariable regions. In 50 μL volumes, the reaction mixture contained 0.3 mg/mL bovine serum albumin (BSA), 250 mM dTNPs, 0.5 mM of each primer, 0.02 U Phusion High-Fidelity DNA Polymerase (Finnzymes OY, Espoo, Finland) and 5× Phusion HF Buffer containing 1.5 mM MgCl_2_. The PCR conditions used were initial denaturation at 95 °C for 5 min, followed by 25 cycles of denaturation (95 °C for 40 s), annealing (2 min), and extension (72 °C for 1 min), with a final extension step at 72 °C for 7 min. For the primer pair, the annealing temperature was adjusted to 55 °C. A QiaQuick PCR purification kit was used to purify the PCR products (QIAGEN, Hilden, Germany). The PCR products were stored at –20 °C until sequencing.

##### PCR amplification of the ITS gene

The ITS gene amplification followed the procedure by Akwa et al. (2020). Primer pairs used were: 5′-TCCGTAGGTGAACCTGCGG-3′ and 5′-TCCGTAGGTGAACCTGCGG-3′. In 50 µL volumes, the reaction mixture contained 1 µL of DNA (final concentration of 10 µM), 250 mM dTNPs, 2 µM of each primer, 0.02 U Phusion High-Fidelity DNA Polymerase (Finnzymes OY, Espoo, Finland) and 5X Phusion HF Buffer containing 1.5 mM MgCl_2_. PCR conditions used were as follows: 34 cycles initial denaturation (98 °C for 1 min), annealing (58 °C for 45 s), and extension (72 °C for 1.5 min), with a final extension step at 72 °C for 10 min. The PCR products were stored at –20 °C until sequencing.

##### Library preparation for sequencing

The extracted DNA was sent to Inqaba Biotechnical Industries (Pty) Ltd., (Pretoria, South Africa) for sequencing. Reads were generated using PacBio single-molecule, real-time (SMRT) technology. To create highly accurate readings (> QV40), raw subreads were processed using the SMRTlink (v7.0.1) circular consensus sequences (CCS) method. The reads were demultiplexed at the sequencing facility prior to analysis.

##### Bioinformatics analyses

The R package DADA2 was used to denoise the reads and identify the relative abundance of each amplicon sequence variant (ASV). Briefly, filtering and removal of low-quality reads were done using the standard parameters. The chimera was removed with the removeBimeraDenovo function. The assignTaxonomy and addspecies functions were used for taxonomic assignment. Amplicon sequencing variants (ASVs) were assigned species-level taxonomy with the full-length trained SILVA138 database for bacterial classification, while the fungal ASVs classification was done using UNITE databases.

##### Exploratory analyses and prediction analyses

Graphical taxa visualisations were performed in R v.3.5.1 and Bioconductor v.3.0 with Phyloseq, and Microbial R packages were used. The *diversity* () function in *vegan* was used to calculate alpha diversity measures. The alpha diversity indices were used to calculate diversity by accounting for dominance and richness. PICRUSt2 was used to predict the microbial function in the microbiota, while subsequent results were visualized and tested for statistical significance in STAMP.

## Results

### Measures of diversity

**Fig. 1:**
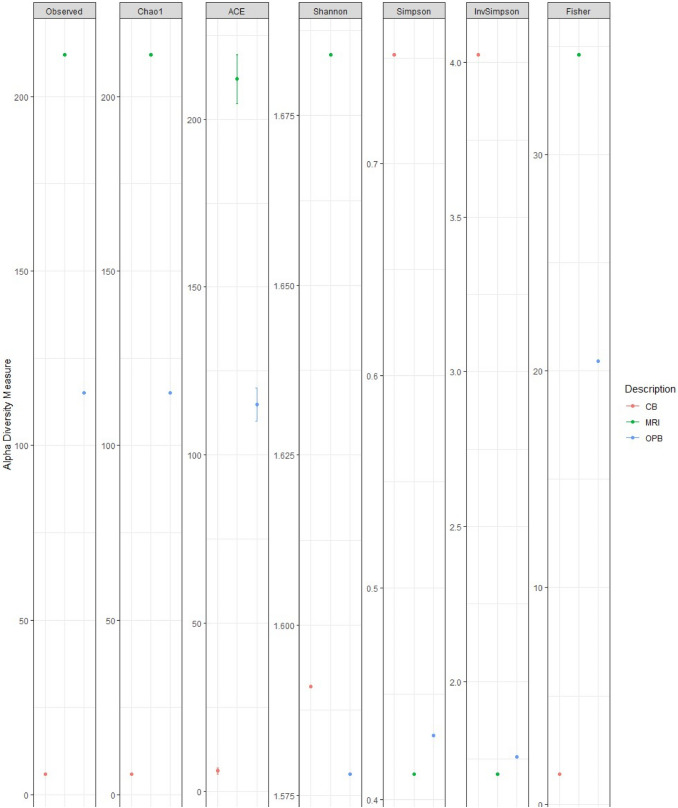
Bacterial alpha diversity metrics based on Chao1, ACE, Shannon, Simpson, InvSimpson, and Fisher’s diversity indices

**Fig. 2:**
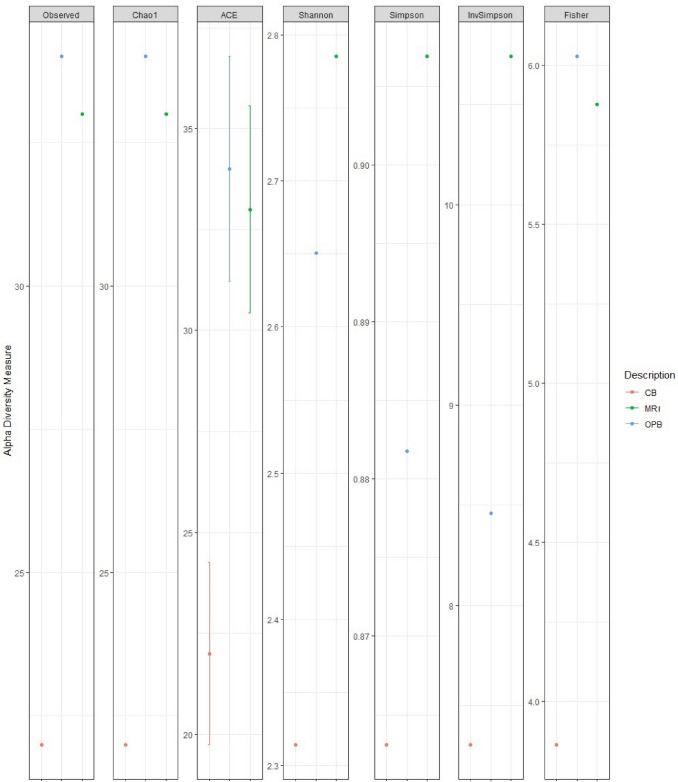
Fungal alpha diversity metrics based on Chao1, ACE, Shannon, Simpson, InvSimpson, and Fisher’s diversity indices

The richness and evenness of bacterial and fungal species in each sample were quantified using Chao1, ACE, Shannon, Simpson, InvSimpson, and Fisher alpha diversity indices. The Chao1 (> 200), ACE (> 200), Shannon (> 1.68), Simpson (< 0.5), and Fisher (> 30) alpha diversity indices showed the highest bacterial species diversity for the MRI sample (Fig. 1**)**. Conversely, these alpha diversity indices showed the lowest bacterial species diversity for the CB. Only the Shannon (< 1.6) index showed the lowest bacterial species diversity for the OPB (Fig. 1). The Chao1 (> 200), ACE (> 200), Shannon (> 1.68), Simpson (< 0.5), and Fisher (> 30) alpha diversity indices showed the highest bacterial species diversity for the MRI sample (Fig. 1). The Chao1 (> 30), ACE (> 30), and Fisher (> 6.0) alpha diversity indices showed the highest fungal species diversity for the OPB sample (Fig. 2**).** Only the Shannon (> 2.7) index showed the highest fungal species diversity for the MRI samples (Fig. 2). The Chao1 (< 25), ACE (< 25), Shannon (< 2.4), and Fisher (< 4.0) alpha diversity indices showed the lowest fungal species diversity for the CB sample (Fig. 2). Conversely, the highest fungal species diversity was observed for the CB sample in the Simpson index (< 0.87) (Fig. 2).

### Microbial community

**Fig. 3:**
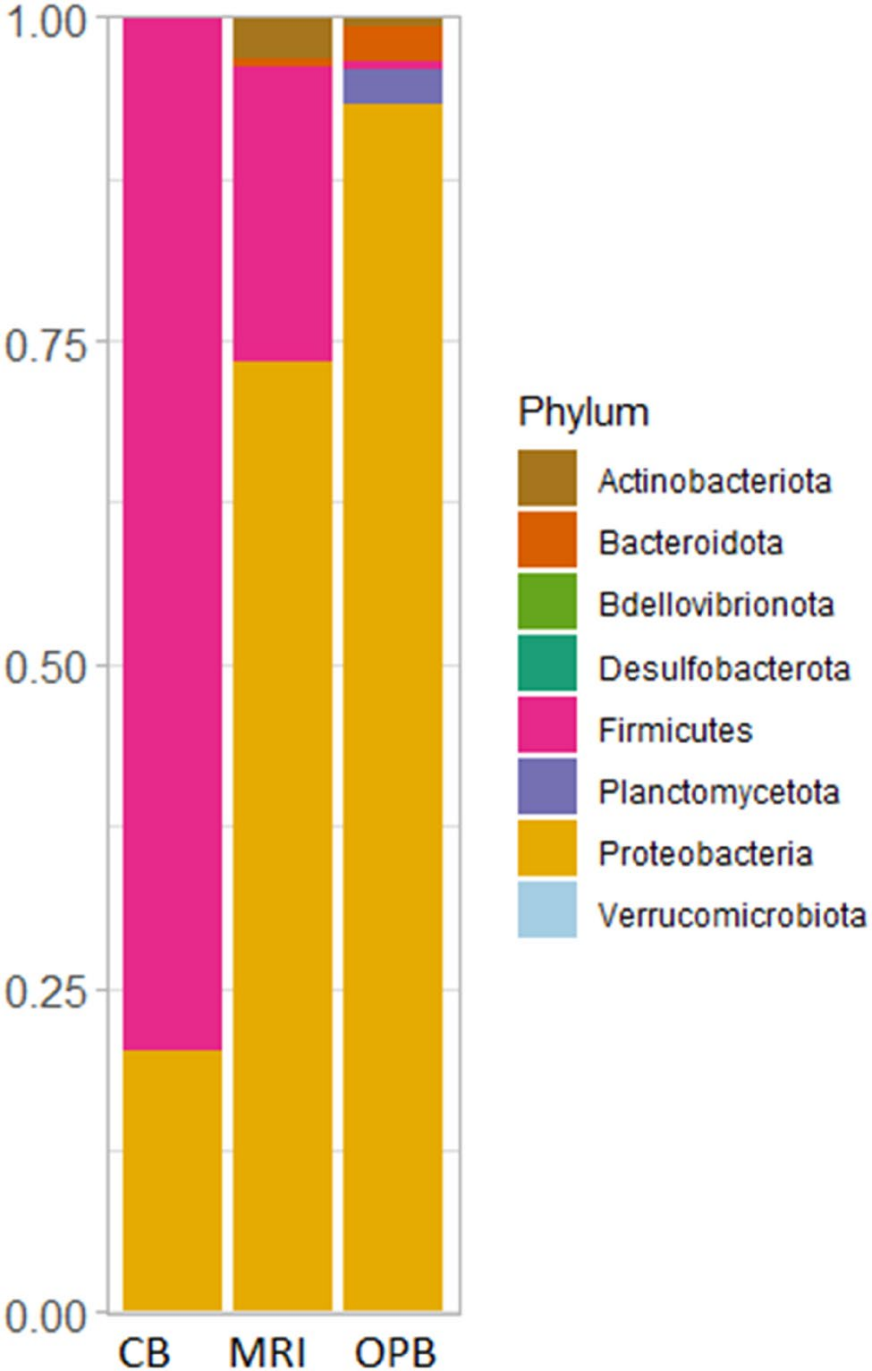
Relative abundance of bacterial communities in the customary beer brew (CB), mixed raw ingredients (MRI), and optimised beer brew (OPB) at phylum level

**Fig. 4:**
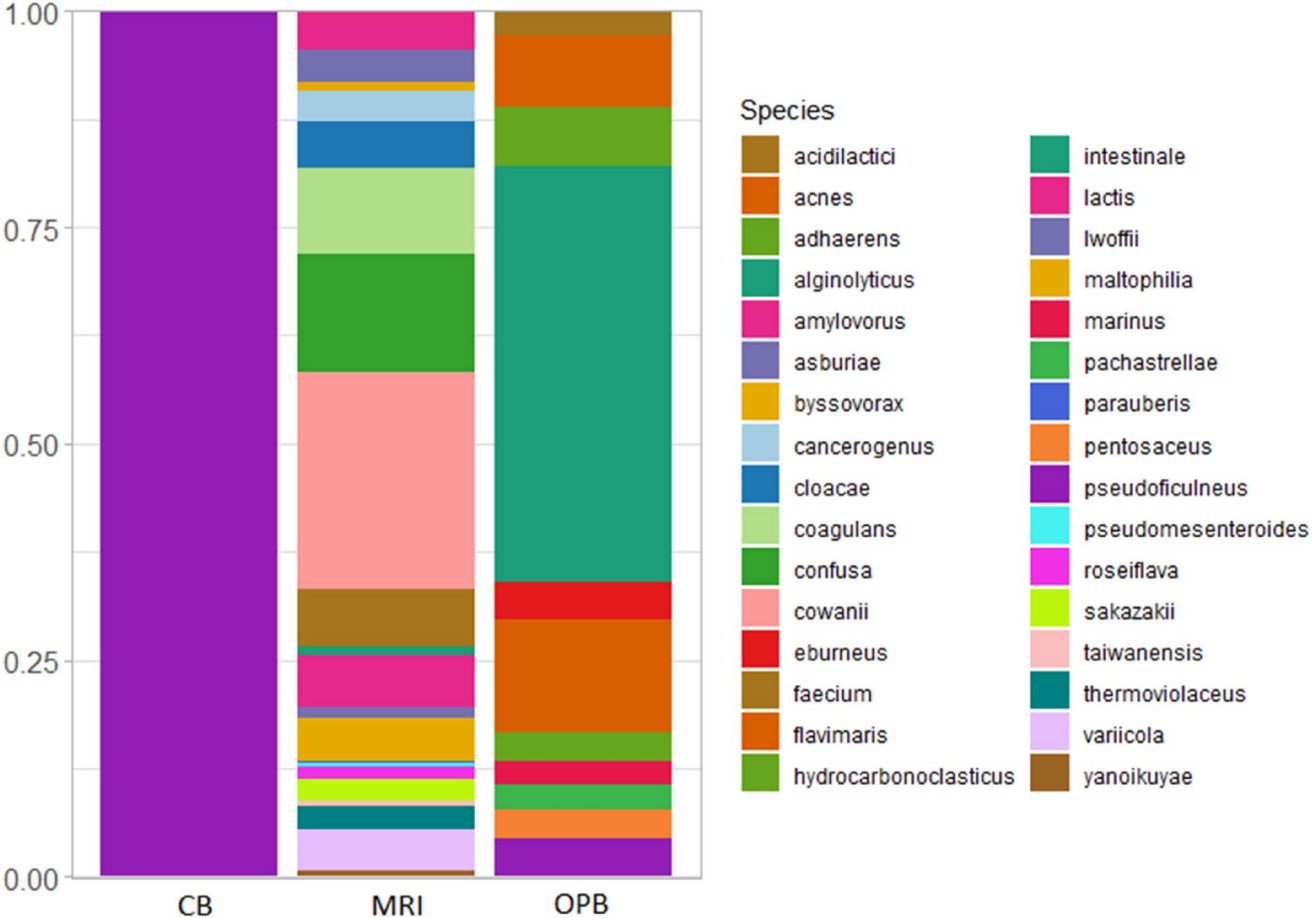
Relative abundance of bacterial communities in the customary beer brew (CB), mixed raw ingredients (MRI), and optimised beer brew (OPB) at species level

At the phylum level, *Proteobacteria* were more abundant in the MRI and OPB, while *Firmicutes* were dominant in the CB (Fig. 3). The dominant classes in the CB, MRI, and OPB samples were Bacilli, γ-proteobacteria, and α-proteobacteria, respectively (Appendix 1). The dominant order greatly varied in the CB (Lactobacillales), MRI (Acetobacterales), and OPB (Rhodobacterales) samples (Appendix 2). At the family level, the dominant groups were Lactobacillaceae, Acetobacteraceae, and Rhodobacteraceae in the CB, MRI, and OPB respectively (Appendix 3). Only the OPB sample contained Vibrionaceae at the family level (Appendix 3). Diversity in the dominant genus was observed in all three samples (Appendix 4). Apilactobacillus, Kosakonia, and Nautella were the dominant classes in the CB, MRI, and OPB respectively (Appendix 4). In addition, each sample contained at least one of the essential lactic acid-producing genera such as *Apilactobacillus*, *Ligilactobacillus*, *Lactiplantibacillus*, *Lactobacillus*, *Lactococcus,* and *Paenibacillus* (Appendix 4) (Quilodrán-Vega et al. 2020). At the species level, *A. pseudoficulneus, Staphylococcus pseudoficulneus*, and *Fructobacillus pseudoficulneus* were the dominant species in the CB sample (Fig. 4). Conversely, *Kosakonia cowanii* was the dominant species in the MRI sample (Fig. 4). *Vibrio alginolyticus* was the dominant species in the OPB (Fig. 4).

**Fig. 5:**
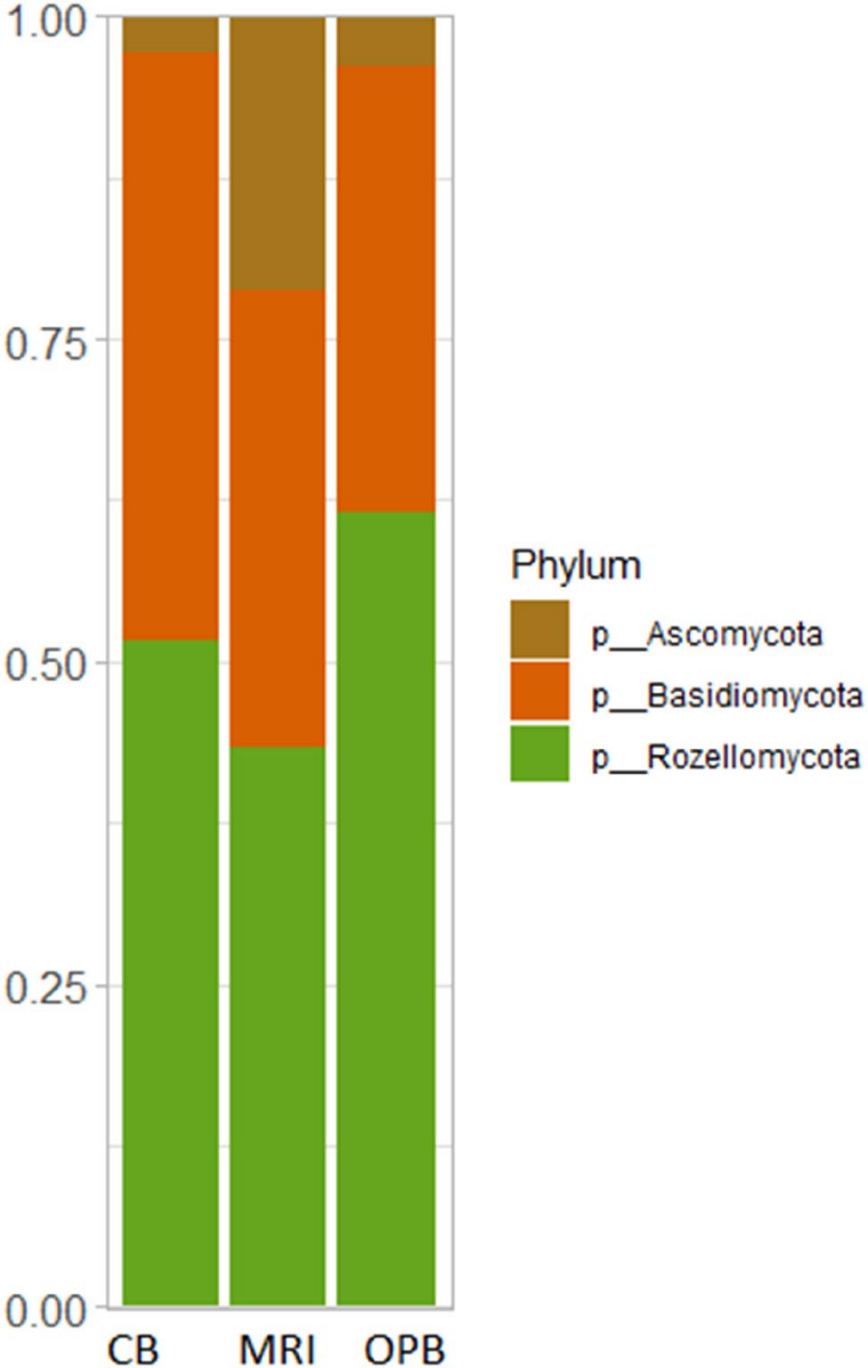
Relative abundance of fungal communities of the customary beer brew (CB), mixed raw ingredients (MRI), and optimised beer brew (OPB) at phylum level

**Fig. 6:**
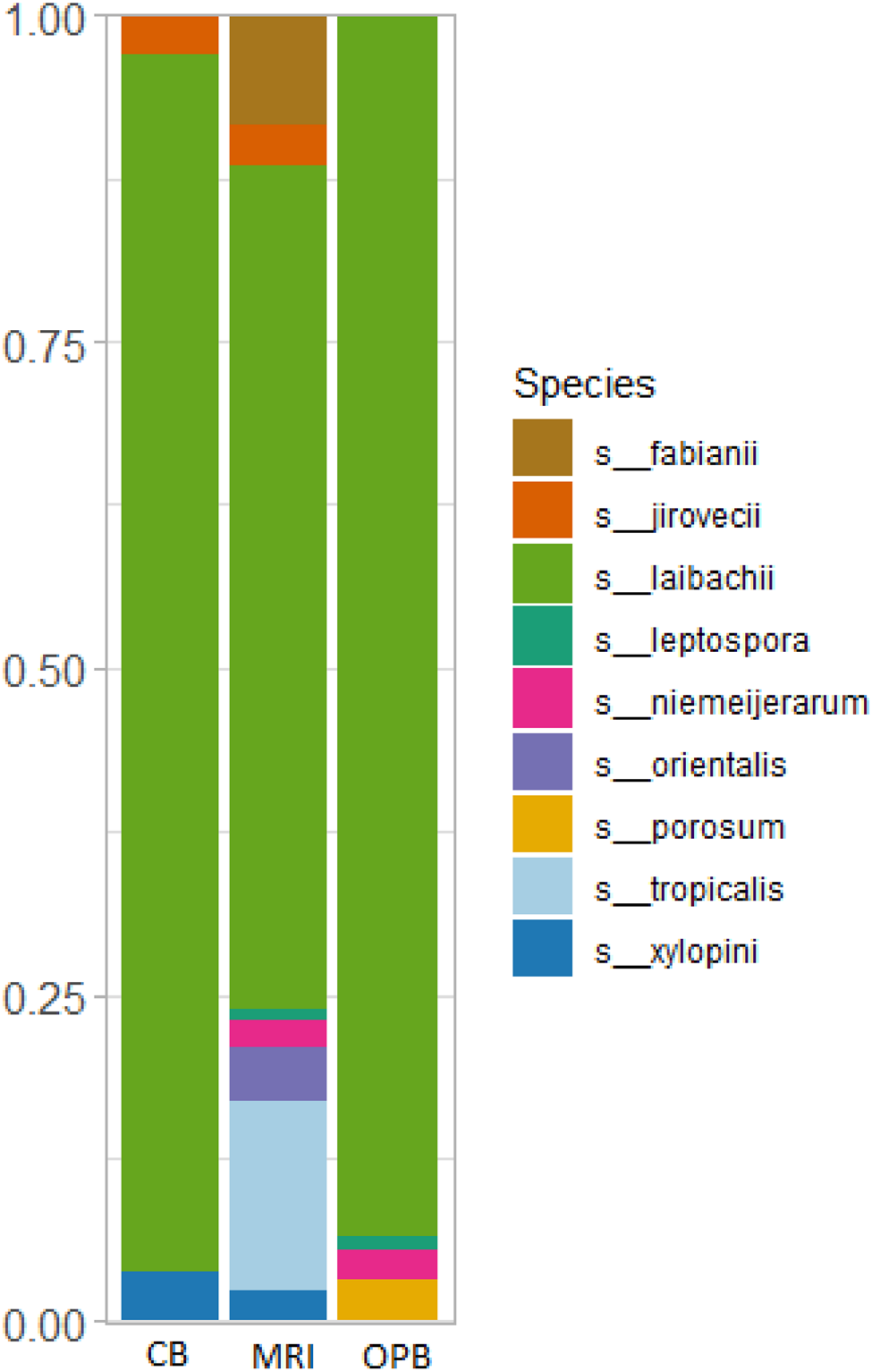
Relative abundance of fungal communities of the customary beer brew (CB), mixed raw ingredients (MRI), and optimised beer brew (OPB) at species level

The total fungal read count was 1233, 1940, and 2037 for MRI, CB, and OPB, respectively. The dominant phylum in all three samples was Rozellomycota, followed by Basidiomycota (Fig. 5). Tremellomycetes were the dominant class in all three samples (Appendix 5). However, this dominance was lower in the MRI compared to the fermented samples, CB, and OPB (Appendix 5). The dominant order in all three samples was Trichosporonales (Appendix 6). At the family level, the MRI and OPB had a greater abundance of fungal communities than the CB (Appendix 7). Trichosporonaceae was the dominant family in all samples, with Trichocomaceae being the second most dominant family group in the fermented samples. The genus *Apiotrichum* was dominant in the MRI, CB, and OPB samples (Appendix 8). The genera *Leucosporidium* and *Penicillium* were also present in the OPB (Appendix 8). At the species level, *A. laibachii* was the dominant group in all three samples (Fig. 6). *A. tropicalis* and *A. orientalis*, were only present in the MRI sample, while *A. porosum* was only present in the OPB sample (Fig. 6).

### Amplicon sequencing variants (ASVs)

**Fig. 7:**
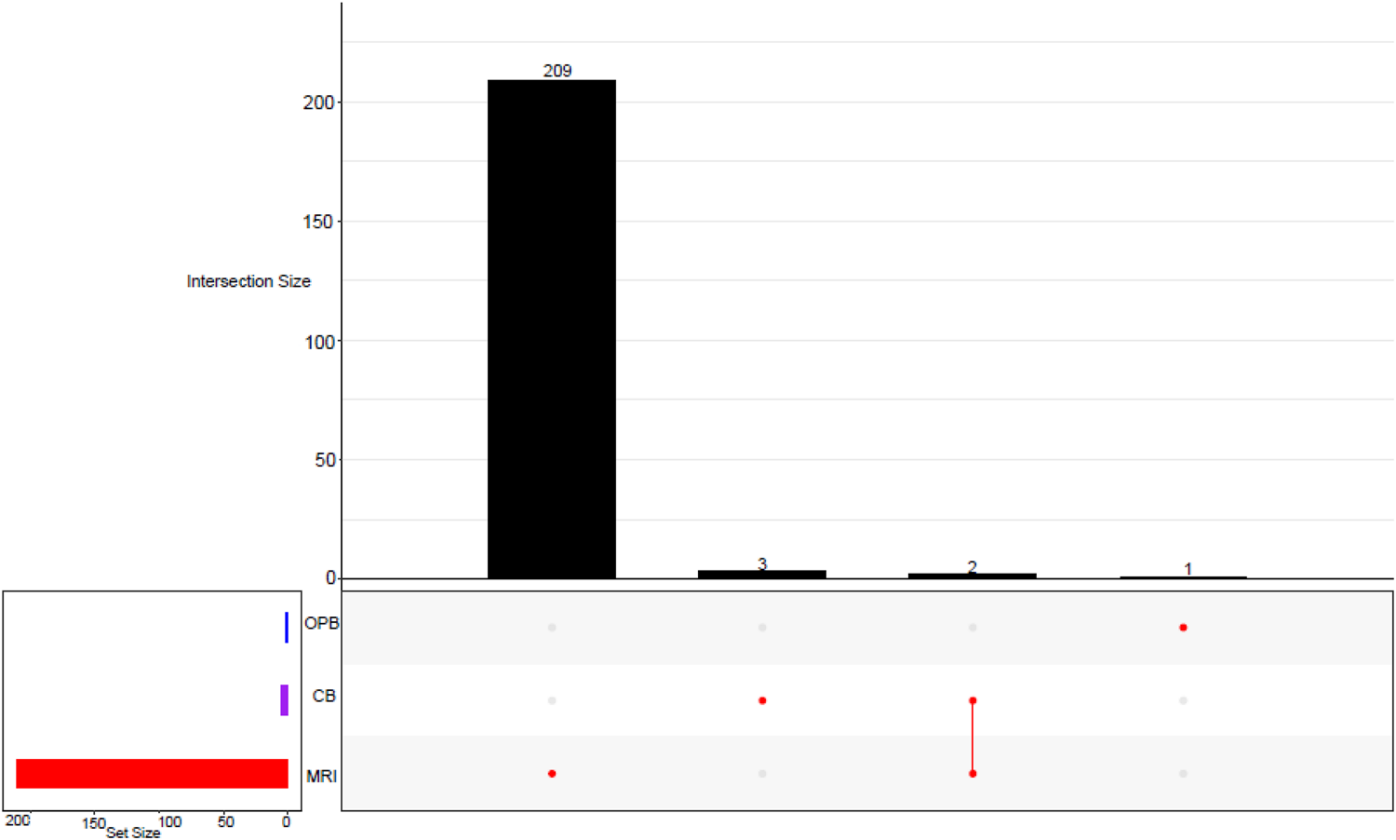
An upset plot showing shared and unique bacterial ASV(s) across different *umqombothi* samples

**Fig. 8:**
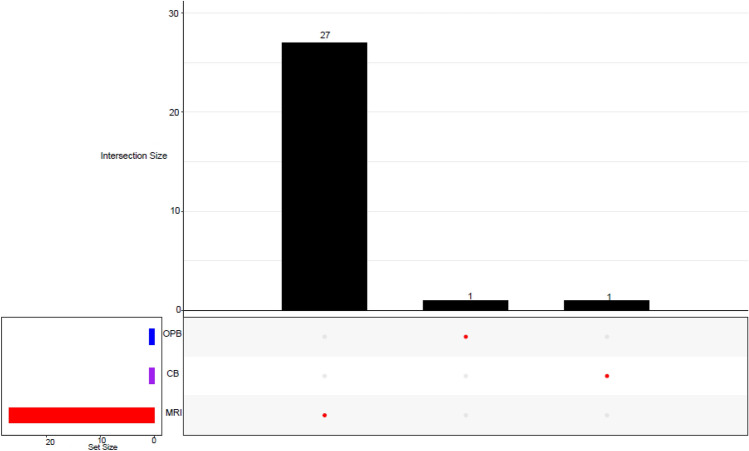
An upset plot showing shared and unique fungal ASV(s) across different *umqombothi* samples

The analyses showed that the bacterial community had higher amplicon sequencing variants (ASVs) (215) (Fig. 7) than the fungal community (29) (Fig. 8). Unique ASVs were highly associated with the MRI group in both bacteria (209) and fungi (27). The high unique ASVs in the MRI in both bacteria (209) and fungi (27) suggest a higher variability (mutations) in the microorganisms within the sample. This result corresponded with the high variability observed in bacterial and fungal communities at the species level (Fig. 4 and Fig. 6). Two bacterial ASVs were shared between CB and MRI, while no shared ASVs were found in the fungal ASVs distribution. The two shared ASVs suggested common genetic features or traits shared by a group of individuals.

### Predictive functional profiles of microbial communities

**Fig. 9:**
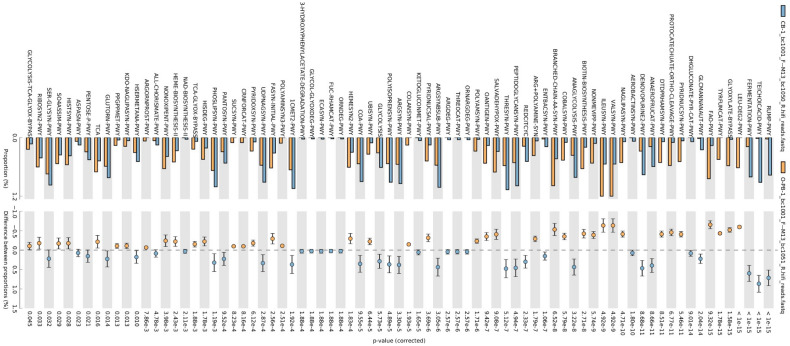
Bar plots showing the mean proportion (%) of significantly different predicted functional compositions between CB and OPB samples

**Fig. 10:**
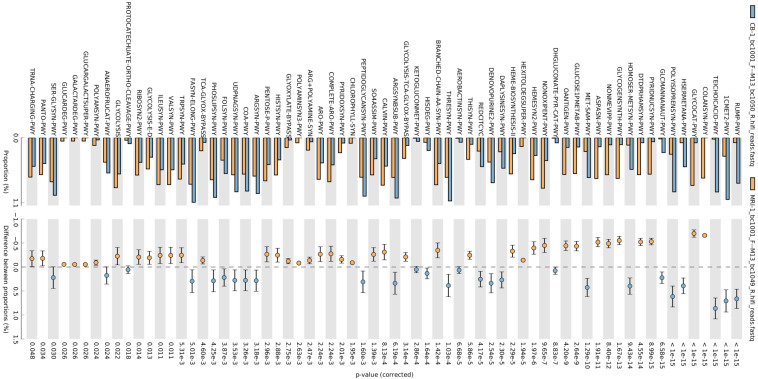
Bar plots showing the mean proportion (%) of significantly different predicted functional compositions between MRI and CB samples

**Fig. 11:**
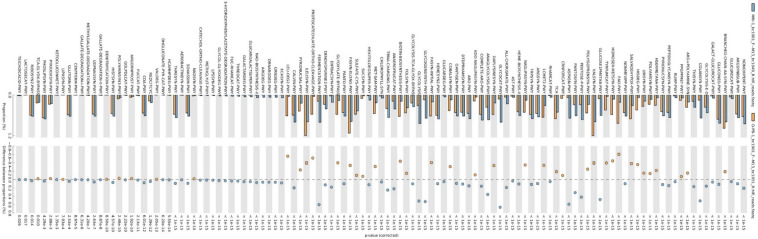
Bar plots showing the mean proportion (%) of significantly different predicted functional compositions between MRI and OPB samples

Phylogenetic Investigation of Communities by Reconstruction of Unobserved States (PICRUSt2) was used to computationally predict the functional composition of metagenomes from marker gene data and a database of reference genomes. These predictions establish connections between metabolic profiles and the phylogenetic structure of microbes. Overall, a total of 120 MetaCyc metabolic pathways were analysed based on the 16S rRNA amplicon data to predict the microbial functional differences between the samples (FDR *p* < 0.05) (Figs. 9 and 10). MetaCyc metabolic pathways for bacteria are shown in Figs. 9, 10 and 11. No significant differences in the metabolic pathways between samples were observed for fungi. The MetaCyc metabolic pathways for fungi are shown in Table 2. The pathways associated with carbohydrate metabolism, amino acid biosynthesis and vitamin biosynthesis were the major enrichments for both bacteria (Figs. 9, 10 and 11) and fungi **(**Table 2**).**

**Table 2:** Predicted functional compositions for fungi

Superclasses	Pathway	*p*-adjusted (FDR) value		
		MRI	CB	OPB
Vitamin biosynthesis	Folate transformations III	0.00	0.15	0.17
Aromatic compound degradation	4-hydroxyphenylacetate degradation	0.00	0.14	0.00
Siderophore and metallophore biosynthesis	Aerobactin biosynthesis	0.00	0.14	0.00
Superpathway of chorismate metabolism	Superpathway of chorismate metabolism	0.00	0.16	0.00
Carbohydrate metabolism	Homolactic fermentation	0.00	0.18	0.00
Carbohydrate metabolism	Glycolysis III (from glucose)	0.00	0.18	0.18
Amine and polyamine biosynthesis superpathways	Superpathway of arginine and polyamine biosynthesis	0.00	0.16	0.00
Amino acid degradation	Superpathway of L-arginine, putrescine, and 4-aminobutanoate degradation	0.00	0.16	0.00
Amino acid biosynthesis	l-arginine biosynthesis I	0.00	0.14	0.17
Amino acid biosynthesis	l-arginine biosynthesis II	0.00	0.15	0.18
Amino acid biosynthesis superpathways	Superpathway of aromatic amino acid biosynthesis	0.00	0.17	0.18
Amino acid biosynthesis superpathways	Superpathway of L-aspartate and L-asparagine biosynthesis	0.00	0.16	0.17
Amino acid degradation	l-arginine degradation II	0.00	0.14	0.00
Cofactor, carrier, and vitamin biosynthesis	Biotin biosynthesis I	0.00	0.16	0.21
Amino acid biosynthesis superpathways	Superpathway of branched chain amino acid biosynthesis	0.00	0.17	0.20
Carbohydrate metabolism	Calvin-Benson-Bassham cycle	0.00	0.19	0.21
Cofactor, carrier, and vitamin biosynthesis	Coenzyme A biosynthesis I	0.00	0.14	0.17
Cofactor, carrier, and vitamin biosynthesis	Superpathway of adenosylcobalamin salvage from cobinamide I	0.00	0.14	0.17
Carbohydrate metabolism	Colanic acid building blocks biosynthesis	0.00	0.16	0.17
Amino acid biosynthesis	Superpathway of aromatic amino acid biosynthesis	0.00	0.18	0.19
Amino-acid-biosynthesis	l-lysine biosynthesis I	0.00	0.15	0.18
Nucleoside and nucleotide biosynthesis	Superpathway of purine nucleotides de novo biosynthesis II	0.00	0.15	0.18
Carbohydrate metabolism	Enterobacterial common antigen biosynthesis	0.00	0.14	0.17
Siderophore and metallophore biosynthesis superpathways	Enterobactin biosynthesis	0.00	0.14	0.00
Fatty acid and lipid degradation	Fatty acid β-oxidation I	0.00	0.28	0.17
Fatty acid biosynthesis	Fatty acid elongation	0.00	0.16	0.24
Fatty acid and lipid biosynthesis	Superpathway of fatty acid biosynthesis initiation	0.00	0.16	0.20
Fermentation of pyruvate Fermentation to alcohols Fermentation to short-chain fatty acids	Mixed acid fermentation	0.00	0.24	0.19
Cofactor, carrier, and vitamin biosynthesis	Superpathway of tetrahydrofolate biosynthesis and salvage	0.00	0.17	0.19
Fucose and rhamnose catabolism	Supepathway of fucose and rhamnose degradation	0.00	0.14	0.00
Carbohydrate metabolism	l-fucose degradation I	0.00	0.14	0.17
Galacturonate and glucuronate catabolism	Superpathway of hexuronide and hexuronate degradation	0.00	0.15	0.00
Carboxylate degradation	d-galactarate degradation I	0.00	0.16	0.00
Carboxylate degradation	d-galacturonate degradation I	0.00	0.14	0.17
Amine and polyamine degradation superpathways	Superpathway of N-acetylglucosamine, N-acetylmannosamine and N-acetylneuraminate degradation	0.00	0.16	0.17
Carboxylate degradation	d-glucarate degradation I	0.00	0.16	0.00
Aldarate catabolism	Superpathway of D-glucarate and D-galactarate degradation	0.00	0.16	0.00
Carbohydrate biosynthesis	Gluconeogenesis I	0.00	0.18	0.19
Carbohydrate metabolism	Glucosee and glucose-1-phosphate degradation	0.00	0.14	0.17
Carbohydrate metabolism	Superpathway of β-D-glucuronosides degradation	0.00	0.15	0.00
Amino acid biosynthesis	l-ornithine biosynthesis I	0.00	0.14	0.17
Carbohydrate metabolism	Glycogen degradation I	0.00	0.19	0.23
Carbohydrate biosynthesis	Glycogen biosynthesis I (from ADP-D-Glucose)	0.00	0.14	0.17
Carbohydrate biosynthesis	Glycogen biosynthesis I	0.00	0.16	0.00
Carbohydrate metabolism	Glycolysis I	0.00	0.19	0.19
Carbohydrate metabolism	Superpathway of glycolysis and the Entner-Doudoroff pathway	0.00	0.17	0.00
Carbohydrate metabolism	Superpathway of glycolysis, pyruvate dehydrogenase, TCA, and glyoxylate bypass	0.00	0.19	0.00
Carbohydrate metabolism	Glyoxylate cycle	0.00	0.16	0.00
Aromatic compound degradation	3-phenylpropanoate and 3-(3-hydroxyphenyl)propanoate degradation to 2-hydroxypentadienoate	0.00	0.15	0.00
Cofactor, carrier, and vitamin biosynthesis	Heme b biosynthesis II	0.00	0.14	0.17
Cofactor, carrier, and vitamin biosynthesis	Heme b biosynthesis II (oxygen-independent)	0.00	0.16	0.19
Carbohydrate metabolism	Superpathway of hexitol degradation	0.00	0.19	0.00
Amino acid degradation	l-histidine degradation I	0.00	0.00	0.17
Amino acid biosynthesis	l-histidine biosynthesis	0.00	0.14	0.17
Amino acid biosynthesis	l-methionine biosynthesis I	0.00	0.14	0.20
Amino acid biosynthesis	l-isoleucine biosynthesis I	0.00	0.18	0.21
Cell structure biosynthesis	Superpathway of (Kdo)2-lipid A biosynthesis	0.00	0.14	0.17
Cofactor, carrier, and vitamin biosynthesis	Superpathway of S-adenosyl-L-methionine biosynthesis	0.00	0.17	0.19
Aldehyde degradation	Superpathway of methylglyoxal degradation	0.00	0.14	0.00
Cofactor, carrier, and vitamin biosynthesis	NAD de novo biosynthesis II (from tryptophan)	0.00	0.17	0.19
Cell structure biosynthesis	Lipid IV_A_ biosynthesis (E. coli)	0.00	0.14	0.17
Secondary metabolite biosynthesis	Methylerythritol phosphate pathway I	0.00	0.14	0.17
Pentose phosphate pathways	Pentose phosphate pathway (non-oxidative branch) I	0.00	0.28	0.26
Amino acid degradation	Superpathway of L-arginine and L-ornithine degradation	0.00	0.16	0.00
Amine and polyamine degradation	Superpathway of ornithine degradation	0.00	0.19	0.00
TCA cycle	TCA cycle IV (2-oxoglutarate decarboxylase)	0.00	0.20	0.00
Fermentation of pyruvate Fermentation to alcohols Fermentation to short-chain fatty acids	Acetylene degradation (anaerobic)	0.00	0.22	0.23
Amino acid biosynthesis	Superpathway of L-lysine, L-threonine and L-methionine biosynthesis I	0.00	0.15	0.18
Carboxylate degradation	Superpathway of N-acetylneuraminate degradation	0.00	0.19	0.19
Carboxylate degradation Fermentation of pyruvate Fermentation to alcohols Fermentation to short-chain fatty acids	Hexitol fermentation to lactate, formate, ethanol and acetate	0.00	0.22	0.00
Cofactor, carrier, and vitamin biosynthesis	Phosphopantothenate biosynthesis I	0.00	0.14	0.17
Cofactor, carrier, and vitamin biosynthesis	Superpathway of coenzyme A biosynthesis I (bacteria)	0.00	0.14	0.17
Pentose phosphate pathways	Pentose phosphate pathway	0.00	0.18	0.20
Cell structure biosynthesis	Peptidoglycan biosynthesis I	0.00	0.15	0.17
Fatty acid and lipid biosynthesis	Superpathway of phospholipid biosynthesis III	0.00	0.22	0.22
Amine and polyamine biosynthesis	Superpathway of polyamine biosynthesis I	0.00	0.18	0.00
Polyprenyl biosynthesis	Polyisoprenoid biosynthesis	0.00	0.14	0.17
Metabolic regulator biosynthesis	ppGpp metabolism	0.00	0.16	0.19
Superpathway of histidine, purine, and pyrimidine biosynthesis	Superpathway of histidine, purine, and pyrimidine biosynthesis	0.00	0.15	0.00
Cofactor, carrier, and vitamin biosynthesis	NAD salvage pathway I (PNC VI cycle)	0.00	0.14	0.00
Cofactor, carrier, and vitamin biosynthesis	NAD de novo biosynthesis I	0.00	0.14	0.17
Cofactor, carrier, and vitamin biosynthesis	Pyridoxal 5′-phosphate biosynthesis I	0.00	0.14	0.00
Carbohydrate metabolism	l-rhamnose degradation I	0.00	0.14	0.00
Cofactor, carrier, and vitamin biosynthesis	Flavin biosynthesis I	0.00	0.14	0.17
Nucleoside and Nucleotide Degradation	Adenosine nucleotides degradation II	0.00	0.23	0.00
Amino acid biosynthesis	Superpathway of L-serine and glycine biosynthesis I	0.00	0.14	0.17
Inorganic nutrient metabolism	Assimilatory sulfate reduction I	0.00	0.19	0.19
Inorganic nutrient metabolism	Superpathway of sulfate assimilation and cysteine biosynthesis	0.00	0.17	0.20
TCA cycle	TCA cycle II	0.00	0.19	0.20
TCA/carbohydrate metabolism	Superpathway of glyoxylate bypass and TCA	0.00	0.18	0.00
Cofactor, carrier, and vitamin biosynthesis	Superpathway of thiamine diphosphate biosynthesis I	0.00	0.14	0.17
Amino acid biosynthesis	l-threonine biosynthesis	0.00	0.17	0.19
Aminoacyl-tRNA charging metabolic clusters	tRNA charging	0.00	0.15	0.18
Amino acid biosynthesis	l-tryptophan biosynthesis	0.00	0.16	0.19
Cofactor, carrier, and vitamin biosynthesis	Superpathway of ubiquinol-8 biosynthesis	0.00	0.15	0.19
Carbohydrate biosynthesis	UDP-N-acetyl-D-glucosamine biosynthesis I	0.00	0.14	0.17
Amino acid biosynthesis	l-valine biosynthesis	0.00	0.18	0.21

*FDR* False Discovery Rate

*p*-adjusted (FDR) < 0.05

#### Predictive functional profiles of bacterial communities

The Kyoto Encyclopedia of Genes and Genomes (KEGG) pathway predictions revealed a high abundance of functional metagenomes in the CB sample (Figs. 9, 10 and 11). The MRI was found to be enriched in predicted superclasses related to amino acid biosynthesis, nicotinamide adenine dinucleotide (NAD) biosynthesis, carbohydrate metabolism, and coenzyme A biosynthesis (Figs. 10 and 11). The most abundant corresponding pathways in the MRI were the branched-chain amino acid (BCAA) biosynthesis super pathway (p = 1 × 10^–15^), NAD de novo biosynthesis I pathway (p = 8.99 × 10^–15^), pentose phosphate pathway (p = 1 × 10^–15^), and coenzyme A biosynthesis I pathway (p = 3.26 × 10^–3^) (Figs. 10 and 11). In contrast, the CB sample was characterized by B-group vitamin biosynthesis, amino acid biosynthesis, and carbohydrate metabolism-related pathways (Figs. 9 and 10). The most abundant pathways in the CB were folate (vitamin B_9_) transformations III (p = 1 × 10^–5^), L-threonine biosynthesis super pathway (p = 1.03 × 10^–4^), and mixed acid fermentation (p = 1 × 10^–5^) (Figs. 9 and 10). Similarly, B-group vitamin biosynthesis, amino acid biosynthesis, and fatty acid and lipid metabolism-related pathways were characteristic of the OPB sample (Figs. 9 and 11). The most abundant pathways in the OPB were biotin (vitamin B_7_) biosynthesis I (p = 1 × 10^–15^), L-valine biosynthesis (p = 1 × 10^–15^), and fatty acid elongation (p = 1 × 10^–15^) (Fig. 9 and 11).

#### Predictive functional profiles of fungal communities

Fungal communities showed a high abundance of functional metagenomes in the MRI sample (*p*-adjusted FDR = 0.00) (Table 2). The MRI was found to be enriched in predicted superclasses related to amino acid biosynthesis, cofactor, carrier, vitamin biosynthesis, and carbohydrate metabolism (Table 2). The corresponding pathways in the MRI were heme b biosynthesis II (*p*-adjusted FDR = 0.00), and coenzyme A biosynthesis I (*p*-adjusted FDR = 0.00) (Table 2). The CB sample was enriched in a predicted superclass related to amino acid degradation, specifically the L-histidine degradation I pathway (*p*-adjusted FDR = 0.00). The OPB sample was enriched in a predicted superclass related to carbohydrate metabolism, amino acid degradation, and carboxylate degradation (Table 2). The corresponding pathways in the OPB were hexitol degradation (*p*-adjusted FDR = 0.00), l-arginine degradation super pathway (*p*-adjusted FDR = 0.00), and d-galactarate/d-glucarate degradation I (*p*-adjusted FDR = 0.00) (Table 2).

## Discussion

### Bacterial and fungal alpha diversity

Chao1 and ACE estimate species richness, while the Shannon and Simpson indices consider both species richness and evenness, with the Simpson also measuring dominance (Kumar et al. 2022). On the other hand, Fisher’s alpha measures comparable diversity between communities (Chen and Shen 2017). The microbial diversity differed significantly between the MRI, CB, and OPB samples. The highest bacterial species diversity was observed in the MRI, which could be associated with differing endogenous microbiota of the raw materials, which was subsequently reduced significantly after fermentation. This finding is consistent with other studies that have found a reduction in bacterial diversity during the fermentation process (Deng et al. 2021; Huang et al. 2022). As a fermentation process proceeds, certain species from dominant bacterial phyla such as Proteobacteria outcompete species from other phyla, as shown by the relative abundance of bacterial communities in this study (Fig. 3 and Fig. 4).

In contrast, the highest fungal species diversity was observed in the OPB sample, suggesting that the fermentation process might have enhanced fungal diversity. The fungi’s relatively high mutation rate allows species to quickly adapt to new and changing microbial environments (Gambhir et al. 2022). Specifically, *Candida* spp. such as *Candida tropicalis* (Fig. 6), *Saccharomyces* spp. and *Hanseniaspora* spp. have been shown to grow well, co-dominate, adapt, and survive stressful alcoholic fermentation (Cureau et al. 2021). The low fungal diversity in the CB, as shown by the Chao1, ACE, Shannon, and Fisher alpha diversity indices, could be due to its limited fermentation time and lower fermentation temperature, factors that drive microbial proliferation (CB fermentation conditions = 24 h at 25 °C compared to OPB fermentation conditions = 25.9 h at 29.3 °C) (Hlangwani et al. 2021a). However, the Simpson index showed the highest fungal species diversity in the CB (< 0.87) (Fig. 2).

### Composition of bacterial communities

The phylum Proteobacteria is the largest and most phenotypically diverse phylogenetic lineage within the domain Bacteria (Kersters et al. 2006a). Because Proteobacteria respond positively to labile carbon compounds, they are often the most prominent keystone taxa in the sorghum rhizosphere networks (Oberholster et al. 2018). The dominance of Firmicutes in the CB is not unusual, as a noticeable shift from Proteobacteria to Firmicutes occurs during fermentation (Mareque et al. 2018). This shift has been demonstrated in the fermentation of the traditional Mexican alcoholic beverage *pulque* and traditional Korean alcoholic beverages *nuruk* and *makgeolli* (Escalante et al. 2008; Jung et al. 2012). Certain, bacterial groups belonging to Proteobacteria and Firmicutes in *Sesotho* beer, a type of *umqombothi* produced in The Kingdom of Lesotho, have been reported in a study by Cason et al. (2020). Their study suggests that the Proteobacteria and Firmicutes present likely originated from the raw materials (maize, sorghum, or malted sorghum) used in the preparation of the *Sesotho* beer, as well as from the brewers themselves (Cason et al. 2020).

The proliferation of Bacilli reduces the growth of unwanted microbes in the beer’s souring mash while producing lactic acid, providing a unique flavour profile and improving the quality and shelf life of the final beer product (Cissé et al. 2019). The classes Acidimicrobiia, Desulfuromonadia, Planctomycetes, and Phycisphaerae were only present in the OPB. Acidimicrobiia are acidophilic chemotrophs with an optimal growth pH of around 2 which possess the ability to oxidize ferrous iron at relatively fast rates (Johnson 2009). Desulfuromonadia, on the other hand, are typical thermophiles that are capable of anaerobic respiration by using sulphur, manganese, iron, trichloroacetic acid, and cobalt as electron acceptors (Brenner et al. 2005). Planctomycetes are biotechnologically important bacteria capable of sterol biosynthesis, nitrogen-fixation, endocytosis, and phagocytosis-like process, anaerobic ammonia oxidation, and sulphate polysaccharides metabolism (Kaboré et al. 2020). The emergence of these classes in the OPB is not surprising, as the sample had a lower pH (Table 1), and an abundance of minerals (iron, nitrogen, sulphur, manganese), and sugar compounds (maltose, glucose, mannitol) (Hlangwani et al. 2021a, b).

Members of Lactobacillales produce proteinaceous bacteriocins, lactic acid and other metabolic products (Katongole 2008) which contribute to the organoleptic and textural profile of *umqombothi*. Acetobacterales are mainly found in sugary raw materials (Kersters et al. 2006b). Fermentation negatively affected the presence of Acetobacterales, as shown by the decrease in relative abundance in the OPB and CB samples (Appendix 2). Acetobacterales achieve optimum growth between pH 5–6.5 (Gomes et al. 2018), and, as a result, the low pH values in the CB (pH = 4.23) and OPB (pH = 3.27) (Table 1) limited their growth. The proliferation of Rhodobacterales in the OPB can be attributed to the sample’s high glutamine content (1.6 g/100 g) (Hlangwani et al. 2021b). The *olsBA* operon in Rhodobacterales contains a gene that codes a homologue of OlsB (*olsB2*), which catalyses glutamine-containing compounds (Geiger et al. 2010). In co-cultures and/or symbiotic systems, certain members of Rhodobacterales have the ability to shift from mutualism to parasitism, increasing the group’s dominance (Wang et al. 2014). To dominate and outcompete other groups for nutrients, Rhodobacterales produces antibiotics and other bactericidal compounds and then utilises the fixed carbon from the lysed cells (Cooper and Smith 2015).

Staphylococcaceae was significantly prevalent in the CB (9.36%) and moderately present in the MRI (0.13%) (Appendix 3). This result is not surprising, as Staphylococcaceae can contaminate raw maize and then rapidly multiply when lactic acid bacteria (LAB) are still in the lag phase and a significant amount of lactic acid is yet to be produced (Tchakounté et al. 2018). At the exponential growth phase of LAB, lactic acid concentration increased with a decrease in pH (Table 1), imparting bacteriostatic and bactericidal effects which subsequently led to a reduction in the prevalence of Staphylococcaceae (Appendix 3). The reduction of Staphylococcaceae during fermentation is a consequence of the longer fermentation period in the OPB sample (time = 25.9 h) compared to the CB sample (time = 24 h). As a result of a higher pH value and a lower lactic acid concentration in the CB compared to the OPB (Table 1), the relative abundance of Staphylococcaceae was higher in the CB sample (Appendix 3). Studies by Ikalafeng (2008) and Lues et al. (2011) have reported the presence of Staphylococcaceae in *umqombothi*. Vibrionaceae are metabolically diverse facultative anaerobes capable of D-glucose, D-fructose, maltose, and glycerol catabolism (Gomez-Gil et al. 2014). It is possible that the reduction in the concentration of the sugars arabitol, fructose, sorbitol, myo-inositol, and sucrose during the fermentation of *umqombothi* (i.e., lower in CB and OPB compared to MRI) was due to the growth of members of this family (Hlangwani et al. 2021b).

Using culture-dependent and culture-independent methods, Mukisa et al. (2012) showed the dominance of LAB in *obushera*, a collective name for popular traditional fermented cereal beverages *obutoko*, *enturire*, *ekitiribita,* and *obuteire* consumed in western, southwestern, and central Uganda. Similarly, a high-throughput sequencing study by Eltayeb et al. (2020) found that the bacterial community in *hulumur* (a Sudanese sorghum-based fermented beverage) was dominated by LAB at the genus level. Currently, not much is known about *A. pseudoficulneus*. However, the majority of *Apilactobacillus* species including *A. pseudoficulneus*, *A. timberlakei*, *A. micheneri*, *A. quenuiae*, and *A. kunkeei* have been described to be heterofermentative, facultatively anaerobic fructophilic lactic acid bacteria (FLAB) which produce lactic acid, and antimicrobial compounds, including bacteriocins (Maeno et al. 2021; Molina et al. 2023). It is possible some of these species were present in the CB but were not detected during sequencing, construction, and/or analysis. Such screening limitations exist in the species-level metagenomic analysis of fermented foods, and thus the use of two or more approaches is recommended (Walsh et al. 2017).

### Composition of fungal communities

Several groups of Rozellomycota, observed in all samples, prefer low pH conditions (3–4) and ambient temperatures, which are simulated in the production of *umqombothi* (Table 1) (Ikalafeng 2008). Tremellomycetes are often isolated from cereal grains such as wheat, rice, maize, and sorghum, and may be found in the mucosal surfaces of approximately 1–10% of the healthy human population (Shoff and Perfect 2021). However, interestingly, they have been the dominant class in some wineries and breweries (Sohlberg et al. 2022). Groups within this class may be extremely harmful or beneficial to humans (Weiss et al. 2014). For example, pathogenic Tremellomycetes may cause white Piedra, an infection of the hair follicles (Shivaprakash et al. 2011), while Tremellomycetes are mycoparasitic fungi that produce a wide range of bioactive, antimicrobial metabolites, carbon-active and proteolytic enzymes, and yeast oils (Abe et al. 2006; Vujanovic 2021). Therefore, Tremellomycetes are useful in the biocontrol of plant-pathogenic fungi such as Smut diseases, *Botrytis*, common bunt, and *Tilletia*, as well as the biodegradation of anti-nutritional compounds such as phenolic compounds (Fonseca et al. 2011; Vujanovic 2021).

Trichosporonales consists of a diverse group of members involved in fermentation processes and biotransformation of biarylic compounds (Takashima et al. 2019; Martínez-Herrera et al. 2021). Saccharomycetales, a common group in maize, were only present in the MRI sample (Sun et al. 2019). Members of Saccharomycetales such as *Schwanniomyces occidentalis*, *Saccharomyces cerevisiae*, and *Candida* are responsible for the fermentation of sugars (Johnson and Echavarri-Erasun 2011). Because Saccharomycetales synthesise butyric acid and caproic acid, they play a key role in beer brewing and flavour formulation (Liu et al. 2021). The mycoparasitic, dimorphic yeast, Leucosporidiales, were only present in the OPB samples (Appendix 6) (van der Klei et al. 2011).

Trichocomaceae are a relatively large family of fungi which are thermotolerant, psychrotolerant, osmotolerant, and xerotolerant, and can grow on diverse substrates (Houbraken and Samson 2011). As a result, Trichocomaceae commonly colonise sorghum grains and a wide range of fermented alcoholic beverages such as Chinese ‘*Baiyunbian*’ liquor, *marcha,* and *thiat* (Moretti and Sarrocco 2016; Hu et al. 2017; Sha et al. 2017). The secondary metabolites (extrolites) secreted by members of Trichocomaceae may be beneficial (e.g., the production of antibiotics such as penicillin and the cholesterol-lowering agent lovastatin) or harmful (e.g., the production of mycotoxins such as patulin, aflatoxins, ochratoxins, and fumonisins) (Moretti and Sarrocco 2016). In addition, members of Trichocomaceae produce organic acids and different enzymes that degrade a range variety of complex biomolecules (Houbraken and Samson 2011).

During fermentation, members of *Leucosporidium* may produce the therapeutic enzyme L-asparaginase (ASNase), coenzymes Q9 and Q10, lipases, and Rhodotorulic acid, which is effective against spore germination of the fungus *Botrytis cinerea* (Sansone et al. 2005; Moguel et al. 2020). In a study by Odhav and Naicker (2002) species of *Penicillium* were isolated from sorghum and sorghum malt grains but *umqombothi* was not studied*.* Adekoya et al. (2018b) reported the presence of 2.30 × 10^5^ CFU/mL *Penicillium* species in *umqombothi* samples. Although members of *Penicillium* produce several mycotoxins, certain species can produce extracellular enzyme systems that alter the chemical composition of the sorghum grains, leading to changes in the flavour and colour of the beer (Odhav and Naicker 2002).

*A. laibachii* has the ability to utilise complex nitrogenous compounds (hydroxyproline, tyramine, uric acid, L-phenylamine and ethylamine), aliphatic lipids, cellobiose, aromatic compounds, ammonium sulphate, and amines as sole sources of carbon and energy (Johnson and Echavarri-Erasun 2011). During fermentation, *A. laibachii* produces the hydrolytic enzymes β-glucosidase, xylanase, and thermostable cellulases which break down insoluble and soluble substrates at elevated temperatures over a wide pH range (Touijer et al. 2019). The use of *Candida tropicalis* as a starter culture in the alcoholic fermentation of *tchapalo*, a traditional sorghum beer of Côte d’Ivoire, resulted in the higher production of organic acids and 2-butanone in pure culture (N’Guessan et al. 2010).

Fructophilic lactic acid bacteria (FLAB) such as *A. porosum* can only metabolize a limited number of carbohydrates (Endo et al. 2018). Generally, they show high metabolic activity in a D-fructose-rich substrate and limited metabolic activity in a D-glucose-rich substrate (Maeno et al. 2021). Furthermore, FLAB are metabolically characterised by a higher production of acetate and not alcohol unlike other groups of heterofermentative LAB (Maeno et al. 2021). This may explain the relatively lower alcohol production (i.e., approximately 4.5–5.5%) reported in a preceding study (Hlangwani et al. 2021a). *A. porosum* has useful applications in alcoholic fermentation, especially in the production of high-value products such as gluconic acid, single-cell oil, and low-alcohol beverages (Gorte et al. 2019). Furthermore, *A. porosum* (syn. *T. porosum*) is highly active against pathogenic *basidiomycetous* and *ascomycetous* fungi, while the fungicidal preparation extracted from the culture broth has been recorded to be effective against *Filobasidiella neoformans* and *Candida albicans* cells (Kulakovskaya et al. 2010). These antifungal and fungicidal cellobiose lipids produced by *T. porosum* have been isolated in Malaysian fermented foods such as *tapai*, *tempeh*, and *miso* (Lim et al. 2011).

### Bacterial and fungal amplicon sequencing variants (ASVs) distribution across the groups

The lower fungal ASVs were expected, as studies show that heterofermentative and homofermentative mesophilic bacteria dominate the mashing process, as well as early stages of the fermentation in the mixed raw ingredients (i.e., MRI) (Tokpohozin 2018; Tyakht et al. 2021). The abundance of bacteria, particularly LAB, is attributed to the sorghum and maize used to prepare *umqombothi* (Liu et al. 2021). Differences in the lactic acid microbiota profiles in the brew can be attributed to the souring temperature (Van Der Walt 1956). Sequentially, lactic acid fermentation is carried out by several LAB, followed by yeast-mediated alcoholic fermentation (Katongole 2008). Cason et al. (2020) found a total of 46 fungal ASVs using deep identical sequencing. A loss in the number of reads and ASVs is often observed post-data analysis due to a lack of adequate fungal ASV classification in environmental studies. In the absence of reference sequences in the database, new species, genera, or families remain unidentified, even at a kingdom classification level (Heeger et al. 2019). As a result, fungal diversity is significantly underestimated and underrepresented (Cason et al. 2020). According to Needham et al. (2017), abundant taxa have a greater chance of displaying rare variants. While microbial variants can have a lower relative abundance, they usually show higher genetic and functional diversity (Cao et al. 2022). Conversely, the two bacterial ASVs shared between the CB and MRI samples (Fig. 7) suggest common genetic traits shared by a group of individuals.

### Predictive functional profiles of bacterial and fungal communities

The metabolic activity observed in the biochemical pathways of bacterial communities is often attributed to LAB cereal-grain fermentation (Kayitesi et al. 2023). Cason et al. (2020), suggested that LAB such as *Lactobacillus*, *Pediococcus*, and *Weisella* in *Sesotho* beer was responsible for the high level of predicted fermentative metabolism. The LAB introduced in the mashing and souring stages of *umqombothi* preparation are highly dependent on the BCAA biosynthesis super pathway for optimal growth, adaptation to the alcoholic conditions, and maintenance of the internal pH (Smid and Hugenholtz 2010). In yeasts such as *S. cerevisiae*, this pathway is responsible for the metabolism of ketobutyrate to isoleucine, and pyruvate to valine, both essential precursors for growth, glucose metabolism, and producing vicinal diketones which impart a butter-tasting flavour (Krogerus and Gibson 2013). This was confirmed by the high enrichment in predicted superclasses related to amino acid biosynthesis and/ or degradation across all three samples (Table 2). The pentose phosphate pathway (PPP) is the catabolic pathway for xylose in naturally xylose-fermenting yeasts, as well as the catabolic pathway for xylulose in *Candida shehatae* and *S. cerevisiae* (Johansson and Hahn-Hägerdal 2002). In the cell, the PPP regulates the concentration of simple sugars and provides precursors for the biosynthesis of aromatic amino acids, nucleic acids, and fatty acids (Kloska et al. 2022). In yeasts such as *S. cerevisiae*, the PPP has a similar contribution to glucose degradation during fermentation (Bertels et al. 2021).

The breakdown of complex sugars such as hexitol (sorbitol and mannitol), l-rhamnose, glycogen, and l-fucose to lactate, formate, ethanol, and acetate (Table 2) were consistent with the findings of a preceding study, which found that there was a reduction in the concentration of the sugars arabitol, fructose, sorbitol, myo-inositol, and sucrose during the fermentation of *umqombothi* (Hlangwani et al. 2021b). In the same study, the MRI, CB, and OPB were shown to be rich in amino acids, and B-group vitamins (Hlangwani et al. 2021b). The fermented samples CB and OPB were particularly rich in valine, and folate (vitamin B_9_) (Hlangwani et al. 2021b). The availability of these compounds drives the metabolic activity of the associated microorganisms (Ledesma-Amaro et al. 2013). Specifically, carbohydrates are substrates used by both bacteria and yeast for biomass formation and the conversion of a spectrum of sugars into alcohol and organic acids (Maicas 2020). In *umqombothi*, amino acids (the main nitrogen source) are an essential component of protein synthesis, production of aromatic compounds, ATP generation, and beer flavour, especially by yeasts such as *S. cerevisiae* (Takagi 2019). Finally, vitamins such as thiamine (B_1_), biotin (B_7_), and pantothenic acid (B_5_) are necessary for yeast growth, enzyme function, and a successful alcoholic fermentation process in *umqombothi* production (Katongole 2008). For example, the absence of thiamine (B_1_) may lead to poor-quality fermentations due to sluggish yeast metabolism as observed in a preceding study (Hlangwani et al. 2021b).

## Conclusion

The culture-independent (i.e., PacBio single-molecule, real-time (SMRT) technology) techniques are highly effective for identifying microbial populations in fermented foods. This advantage was demonstrated through the abundance of data detailing the bacterial and fungal profiles in *umqombothi*. A mixture of harmful and beneficial microorganisms was observed in the MRI, CB, and OPB samples. The microbial diversity differed significantly between the MRI, CB, and OPB. The highest bacterial species diversity was observed in the MRI, while the highest fungal species diversity was observed in the OPB. The dominant bacterial species in the MRI, CB, and OPB were *Kosakonia cowanii*, *Apilactobacillus pseudoficulneus*, and *Vibrio alginolyticus*, respectively. The dominant fungal species across the MRI, CB, and OPB samples was *Apiotrichum laibachii.* The predicted functional annotations revealed fundamental differences in the pathways of the microbes in the fermented and unfermented samples. The most abundant pathways in the MRI were the branched-chain amino acid biosynthesis super pathway (p = 1 × 10^–15^), and the pentose phosphate pathway (p = 1 × 10^–15^). The CB sample was characterised by folate (vitamin B_9_) transformations III (p = 1 × 10^–5^), and mixed acid fermentation (p = 1 × 10^–5^). Biotin (vitamin B_7_) biosynthesis I (p = 1 × 10^–15^), and L-valine biosynthesis (p = 1 × 10^–15^) were characteristic of the OPB sample. Thus, this study contributes towards the advancement of our understanding of microbial communities in *umqombothi* through culture-independent techniques, offering insights into the diversity, dominant species, and functional pathways of the fermentation microbes. Furthermore, these findings can be used in assessing potential starter cultures for commercial production, upon which when adopted can facilitate the production of a quality and safe product. Further studies are however still required to isolate these potential starter cultures, investigate their genomic profile, utilize them for *umqombothi* processing and assess the overall quality of the resultant beverage.

### Supplementary Information

Below is the link to the electronic supplementary material.

Supplementary file1 (DOCX 173 KB)

## Data Availability

The nucleotide sequence data reported are available in the NCBI GenBank databases under the BioSample accession numbers SAMN26992645–SAMN26992650. BioProject ID: PRJNA845913.
