# Supplementary material for: Analysis of the bacterial and fungal populations in South African sorghum beer (umqombothi) using full-length 16S rRNA amplicon sequencing
Source: World J Microbiol Biotechnol. 2023 Oct 21;39(12):350. doi: 10.1007/s11274-023-03764-4 (PMC10589195; doi:10.1007/s11274-023-03764-4)
Supplement: Supplementary file 1 — Supplementary file1 (DOCX 173 KB) [file 11274_2023_3764_MOESM1_ESM.docx]

**Supplementary material**


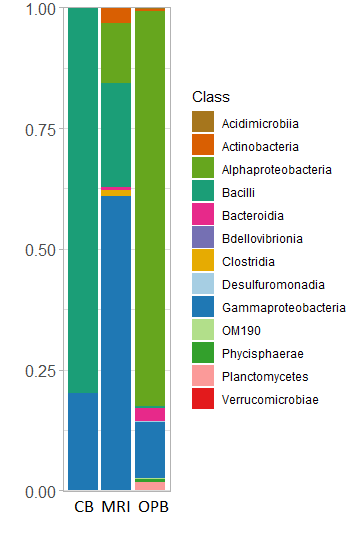


**Appendix 1**. Relative abundance of bacterial communities in the customary beer brew (CB), mixed raw ingredients (MRI), and optimised beer brew (OPB) at class level.


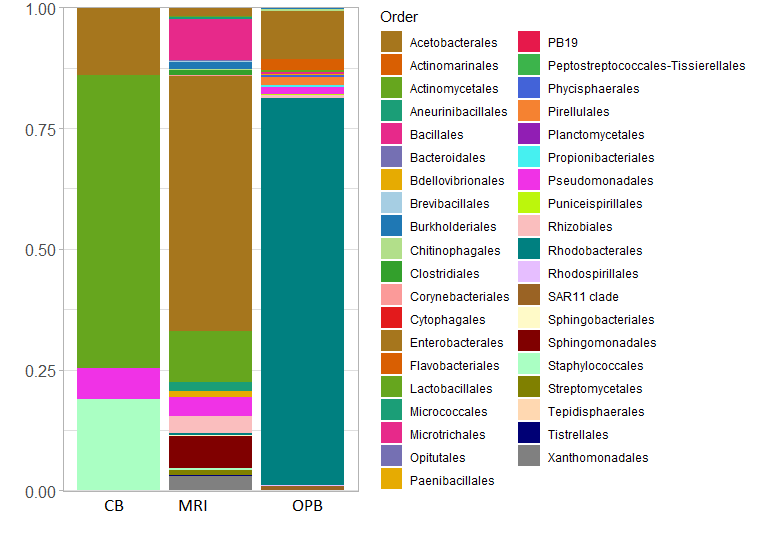


**Appendix 2**. Relative abundance of bacterial communities in the customary beer brew (CB), mixed raw ingredients (MRI), and optimised beer brew (OPB) at order level.


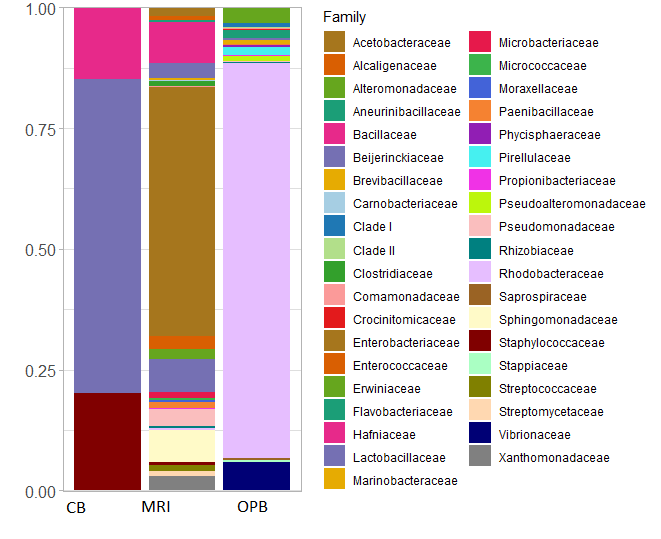


**Appendix 3**. Relative abundance of bacterial communities in the customary beer brew (CB), mixed raw ingredients (MRI), and optimised beer brew (OPB) at family level.


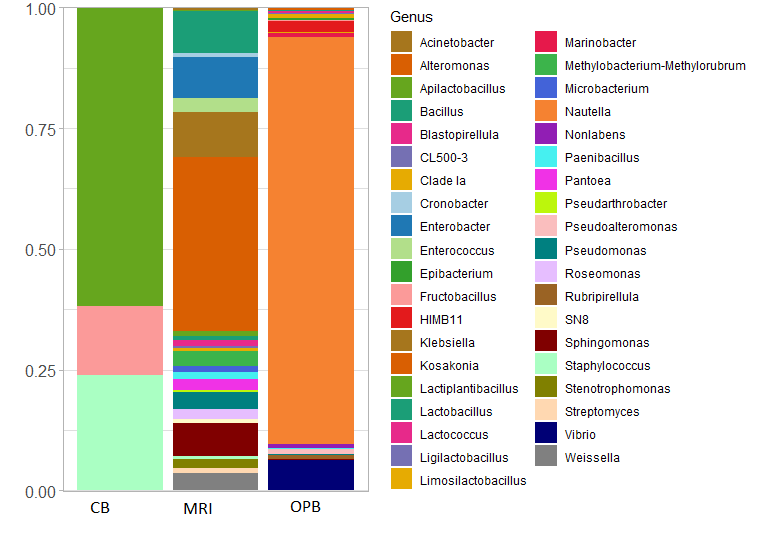


**Appendix 4**. Relative abundance of bacterial communities in the customary beer brew (CB), mixed raw ingredients (MRI), and optimised beer brew (OPB) at genus level.


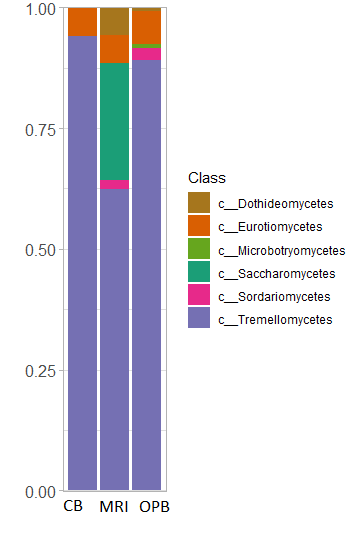


**Appendix 5**. Relative abundance of fungal communities of the customary beer brew (CB), mixed raw ingredients (MRI), and optimised beer brew (OPB) at class level.


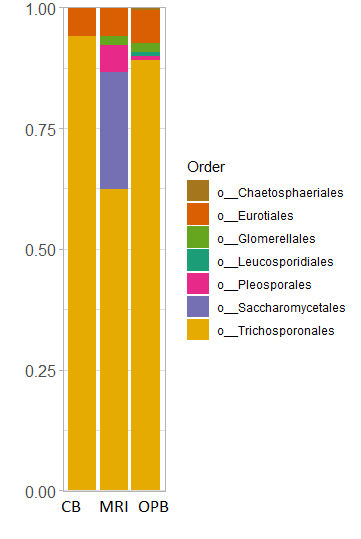


**Appendix 6**. Relative abundance of fungal communities of the customary beer brew (CB), mixed raw ingredients (MRI), and optimised beer brew (OPB) at order level.


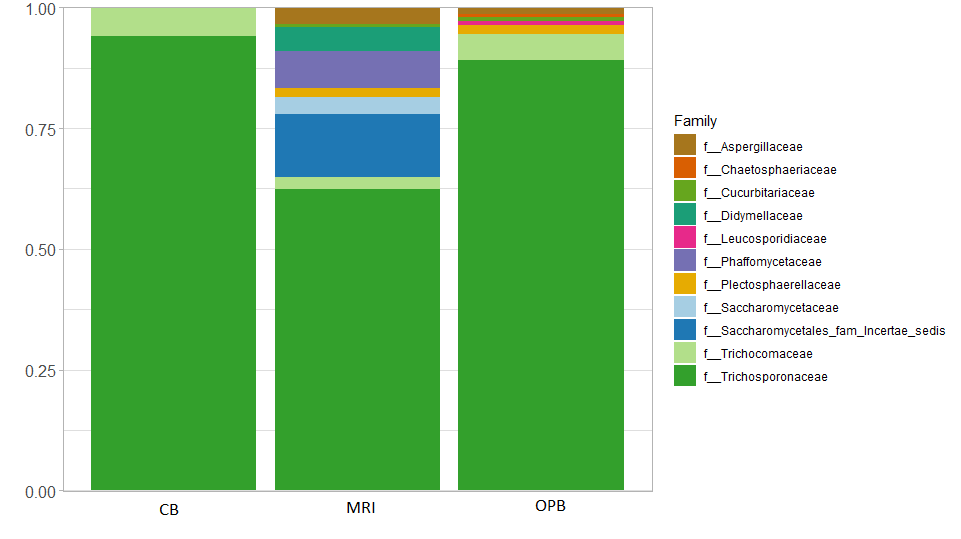


**Appendix 7**. Relative abundance of fungal communities of the customary beer brew (CB), mixed raw ingredients (MRI), and optimised beer brew (OPB) at family level.


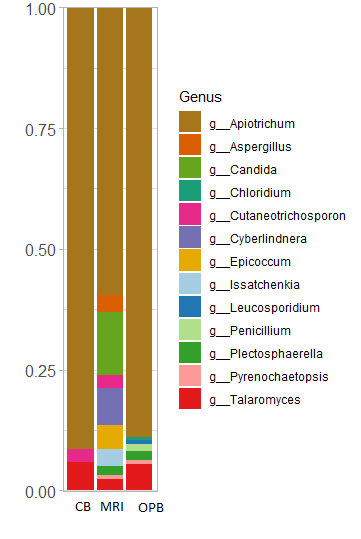


**Appendix 8**. Relative abundance of fungal communities of the customary beer brew (CB), mixed raw ingredients (MRI), and optimised beer brew (OPB) at genus level.
